# Improving mealtimes for people with dementia: Feasibility study

**DOI:** 10.1002/alz.084912

**Published:** 2025-01-09

**Authors:** James Faraday, Annette Hand

**Affiliations:** ^1^ The Newcastle upon Tyne Hospitals NHS Foundation Trust, Newcastle upon Tyne United Kingdom; ^2^ Northumbria University, Newcastle United Kingdom

## Abstract

**Background:**

Mealtimes are a fundamental part of life; eating and drinking well is vital for health well‐being. People living with dementia are at increased risk of eating and drinking difficulties, and may experience difficulties at mealtimes due to the cognitive component of this condition. Such difficulties are prevalent in care homes, where people living with dementia are often dependent on carers at mealtimes. Mealtime care for this population can be variable, with an unhelpful focus on process and systems. This study will test the feasibility of an evidence‐based training intervention designed to improve mealtimes for people with dementia.

**Methods:**

The study builds on previous work in which a care homes training intervention was co‐developed with experts by experience, informed by systematic review data and ethnographic fieldwork. Up to 30 staff are being recruited to participate in the study and receive the training intervention, across several care homes. Outcome measures are the Sense of Competence in Dementia Care Staff (SCIDS) scale, and Confidence in Mealtime Care questionnaire, which has been developed with experts by experience. Focus groups are also being used to gather participants’ views on the intervention. Data is being analysed using descriptive statistics and thematic analysis. A patient and public involvement (PPI) advisory group, comprising care home residents and staff, has shaped the research process throughout.

**Results:**

This feasibility study is essential to ensure the mealtime care training intervention is acceptable and practicable in care homes, and fit for purpose. The PPI advisory group have provided significant input into the design of the study, for example highlighting the importance of a skilled facilitator to deliver training, and advising on how best to measure staff confidence/knowledge. Results of the feasibility study are due later this year.

**Conclusion:**

This study is designed to address uncertainties about a future randomised control trial (RCT) of a mealtime care training intervention in the care homes, including recruitment, attendance rate, and compliance with training. The RCT will also consider resident outcomes such as incidence of aspiration pneumonia, nutritional outcomes, and quality of life outcomes, as well and other outcomes including staff wellbeing.

